# Les facteurs prédictifs de malignité dans la prise en charge des tumeurs parotidiennes: à propos de 76 cas

**DOI:** 10.11604/pamj.2016.23.112.8404

**Published:** 2016-03-16

**Authors:** Brahim Bouaity, Youssef Darouassi, Mehdi Chihani, Mohamed Mliha Touati, Haddou Ammar

**Affiliations:** 1 Service d'Oto-Rhino-Laryngologie, Hôpital Militaire Avicenne, Marrakech, Maroc

**Keywords:** Tumeurs parotidiennes, facteurs prédictifs de malignité, parotidectomie, Parotid tumors, predictors of malignancy, parotidectomy

## Abstract

La pathologie tumorale de la glande parotide est complexe et pose un problème diagnostique et thérapeutique. Une bonne analyse des facteurs prédictifs de malignité de ces tumeurs parotidiennes semble actuellement nécessaire en vue d'une meilleure planification thérapeutique. Le but de ce travail est d’étudier les facteurs prédictifs de malignité dans les tumeurs parotidiennes à travers une étude rétrospective sur 76 cas de tumeurs de la parotide traités au service d'Oto-Rhino-Laryngologie et de Chirurgie Cervico-Faciale de l'hôpital militaire Avicenne de Marrakech entre janvier 2000 et décembre 2012. Il s'agit de 40 femmes et 36 hommes. L’âge moyen était de 44 ans pour les tumeurs bénignes alors qu'il était de 50 ans pour les tumeurs malignes. Le délai moyen de consultation était de 24 mois pour les tumeurs bénignes et de 16 mois pour les tumeurs malignes. La tuméfaction de la région parotidienne a été un signe révélateur constant chez tous les malades. La malignité est évoquée cliniquement devant la douleur, la paralysie faciale, la fixité par rapport au plan superficiel ou profond et la présence d'adénopathie. L'IRM constitue désormais l'examen de choix dans l'exploration des masses tumorales parotidiennes avec une bonne valeur diagnostique de malignité ou de bénignité. La cytoponction à l'aiguille fine n'a pas de valeur que si elle est positive. La parotidectomie exploratrice avec examen anatomopathologique extemporané demeure la clé du diagnostic positif. Les tumeurs parotidiennes bénignes représentent l'entité la plus fréquente (80%) et l'adénome pléomorphe demeure le type histologique prédominant (61%). Quant aux tumeurs malignes, elles sont plutôt rares, dominées essentiellement par les carcinomes muco épidermoides (6,5%). Le traitement chirurgical est l'option de choix souvent associée à un curage ganglionnaire et une radiothérapie pour les tumeurs malignes. La paralysie faciale est la complication la plus fréquente de la chirurgie parotidienne.

## Abstract (en)

Salivary gland tumor pathology is complex and poses a diagnostic and therapeutic problem. A good analysis of predictive factors for malignancy in parotid tumors seems currently necessary for better therapeutic planning. The aim of this study was to investigate the predictive factors for malignancy in parotid tumors through a retrospective study of 76 cases of parotid tumor treated in a service of Otorhinolaryngology and Cervico Facial Surgery of Avicenne military hospital of Marrakech between January 2000 and December 2012. The study involved 40 women and 36 men. The average age was 44 years for benign tumours whereas it was 50 years for malignant tumours. The median of consultation time was 24 months for benign tumors and 16 month for malignant tumours. Swelling in the area of the parotid was always a patient detecting sign. Malignancy is clinically suspected based on pain, facial paralysis, surface structure and deeper structure fixity and on the presence of adenopathy. MRI has become the methodology of choice for evaluating parotid tumors due to its good diagnostic value in the assessment of benignity and malignancy. Fine needle aspiration biopsy has no value unless it is positive. Explorative parotidectomy with extemporaneous anatomopathological examination remains the key to positive diagnosis. Parotid benign tumors represent the most frequent entity (80%) and pleomorphic adenoma remains the predominant histologic type (61%). With regard to malignant tumors, they are rare, mainly dominated by mucoepidermoid carcinomas (6,5%). Surgical treatment is the first choice and it is often associated with lymph node dissection and radiation therapy for malignant tumors. Facial paralysis is the most common complication of parotid surgery.

## Introduction

La pathologie tumorale des glandes salivaires demeure relativement rare représentant 3 à 4% de l'ensemble des tumeurs de la tête et du cou. Leur localisation parotidienne est prédominante [1, 2]. Les formes bénignes prédominent avec comme chef de fil l'adénome pléomorphe. Les données de l'examen clinique, associées à celles de l'imagerie et de la cytoponction constituent des arguments solides en faveur de la malignité ou non de ces tumeurs, mais l'examen anatomopathologique reste l’élément clé du diagnostic positif. La chirurgie constitue le traitement de choix des tumeurs bénignes, le geste chirurgical repose sur une parotidectomie totale conservatrice du nerf facial, ou superficielle « exo-faciale » selon les indications. En cas de tumeur maligne, la parotidectomie totale est la règle associée ou non à un curage ganglionnaire et/ou à une radiothérapie. L'objectif de ce travail est d’étudier les facteurs épidémiologiques, cliniques et paracliniques présomptifs de malignité dans les tumeurs parotidiennes en vue d'une meilleure planification thérapeutique tout en rapportant, à travers une série de 76 cas, l'expérience de notre service dans la prise en charge de cette pathologie.

## Méthodes

Il s'agit d'une étude rétrospective réalisée au service d'Oto-rhino-laryngologie à l'Hôpital Militaire Avicenne de Marrakech couvrant une période de 12 ans, et portant sur 76 cas de tumeurs parotidiennes. Nous avons inclus dans notre étude les dossiers des patients ayant présenté une tumeur parotidienne primitive confirmée à l'examen anatomopathologique définitif. Les tumeurs extra parenchymateuses ont été exclues de notre étude. Pour chaque dossier, nous avons étudié l’âge, le sexe, les antécédents pathologiques, les signes d'appel ayant amené les patients à consulter, ainsi que leur délai d'apparition. Les données de l'examen clinique et des examens radiologiques et cytologiques réalisés ont été également étudiées.

## Résultats

**Figure 1:**
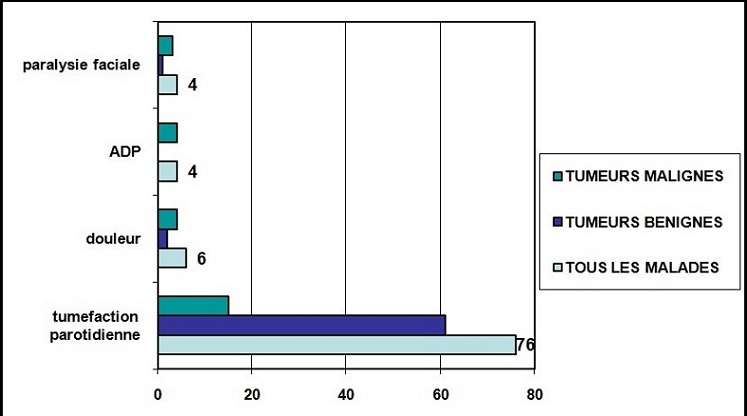
Signes révélateurs des tumeurs parotidiennes

Notre série est composée de 76 patients; 36 hommes et 40 femmes. Le sex-ratio (F/H) était de 1.34 pour les tumeurs bénignes et de 0.5 pour les tumeurs malignes. L’âge moyen de nos patients était de 45 ans. Un pic de fréquence a été noté pour la 4ème décade de vie. Nous n'avons pas trouvé d'antécédents particuliers chez nos patients. Le délai moyen de consultation était de 24 mois pour les tumeurs bénignes et de 16 mois pour les tumeurs malignes. La tuméfaction parotidienne a été le signe révélateur commun chez tous nos patients. La Figure 1 résume les signes révélateurs retrouvés dans notre série et les Tableau 1 et Tableau 2 montrent la corrélation entre la nature histologique et la consistance et la mobilité de la tuméfaction à l'examen clinique. L'examen de l'orifice du canal de Sténon ainsi que celui des glandes sous mandibulaires était sans anomalies dans tous les cas. Sur le plan para clinique, l’échographie cervicale a été réalisée chez 60 patients. Elle avait montré des signes échographiques suspects de malignité dans 6 cas (masses hétérogènes mal limitées). Le scanner réalisé chez 30 patients (53%) a évoqué une tumeur maligne chez 10 patients en montrant un aspect hétérogène de la tumeur avec des limites irrégulières et un rehaussement massif à l'injection du produit de contraste. L'IRM réalisée dans 23 cas (30%) a évoqué une tumeur bénigne chez 10 patients devant des formations nodulaires bien limitées et rehaussées de façon homogène à l'injection du gadolinium. Par ailleurs, un processus tissulaire hétérogène, mal limité, en hypo signal T2, développé aux dépend des deux lobes superficiel et profond a été retrouvé chez 10 patients avec refoulement en dedans de la graisse para pharyngée et extension au pavillon de l'oreille dans un seul cas. Une formation hétérogène à centre nécrosé au dépend du lobe profond avec adénopathies sous digastriques dans deux cas. La cytoponction à l'aiguille fine a été réalisée chez 12 patients (16%); elle était non concluante dans 3 cas, bénigne dans 7 cas et maligne dans 2 cas. Tous nos patients ont été opérés, la parotidectomie a été exofaciale chez 37 patients (48%) et totale dans 39 cas (52%). Le sacrifice du nerf facial a été effectué chez un seul patient. Un geste ganglionnaire a été associé, en cas de tumeurs malignes, dans 32% des cas. L'examen histologique extemporané a été réalisé de façon systématique chez tous les patients de notre série, il a été en faveur d'une tumeur bénigne dans 61 cas (80%) et en faveur d'une tumeur maligne pour 15 patients (20%). L'examen définitif a conclu à 47 cas d'adénomes pléomorphes (61%). Concernant les tumeurs malignes, Le carcinome muco épidermoide est le type dominant. (Tableau 3). Parmi les complications post opératoires, on avait noté 2 cas d'infection de la plaie opératoire, 3 cas de parésie faciale transitoire ayant régressé sous traitement médical avec kinésithérapie et 2 cas de paralysie faciale définitive. Le syndrome de Frey est survenu chez 2 patients. Un bilan d'extension a été réalisé chez tous les patients ayant des tumeurs malignes et n'a pas montré d'autres localisations tumorales. 12 patients ont subi une radiothérapie post-opératoire et un autre a subi une chimiothérapie. Après un recul de 3 ans, 6 de nos patients ont été perdus de vue. Sur les 70 malades restants, l’évolution a été marquée par 2 échecs thérapeutiques chez 2 patients qui ont eu une reprise évolutive de leur maladie avant 12 semaines de la fin de la radiothérapie. Une seule récidive a été notée, c’était une femme présentant un carcinome à cellules claires, elle a été adressée pour radiothérapie et décédée 5 mois plus tard, une bonne évolution a été notée dans le reste des cas.

**Tableau 1:** Corrélation entre la nature histologique et la consistance de la tumeur

Consistance de la tumeur	Tous les malades	Tumeurs bénignes			Tumeurs malignes	
	Nombre	%	Nombre	%	Nombre	%
Ferme	55	72%	49	81%	6	40%
Dure	16	22%	8	13%	8	54%
Rénitente	5	6%	4	6%	1	6%

**Tableau 2:** Étude de la corrélation entre la nature histologique et la mobilité par rapport aux plans de voisinage

Mobilité		Tous les patients		Tumeurs bénignes		Tumeurs malignes	
		Nombre	%	Nombre	%	Nombre	%
Peau	mobile	72	94%	61	100%	11	73%
	Fixe	4	6%	0	0%	4	27%
Plan profond	mobile	61	80%	52	86%	9	60%
	Fixe	15	20%	9	14%	6	40%

**Tableau 3:** Résultats histologiques des tumeurs parotidiennes de notre série

	Type histologique	Nombre de cas	Pourcentage
Tumeurs bénignes (61 cas= 80%)	Adénome pléomorphe	47	61%
	Tumeur de Warthin	7	9%
	Adénome à cellules basales	3	4%
	Cystadénomes	2	2.5
	Papillome canalaire	2	2.5
Tumeurs malignes (15 cas= 20%)	Carcinome muco épidermoide	5	6.5%
	Carcinomes adénoide kystique	4	5%
	Carcinome à cellules claires	3	4%
	Carcinome à cellules acineuses	2	2.5%
	LMNH	1	1.5%
	Adénocarcinome	1	1.5%

## Discussion

Les tumeurs de la parotide représentent une entité relativement rare, constituant environ 3% des tumeurs cervico-faciales et 0,6% des tumeurs humaines [1, 2]. Cependant, leur étude suscite un grand intérêt vue les diverses possibilités d'investigations para cliniques, la grande diversité histologique et les traitements souvent complexes surtout sur le plan chirurgical. Pour Lin, sur une série de 271 patients, le sex-ratio (H/F) était de 1 pour les tumeurs bénignes et de 3 pour les tumeurs malignes; l’âge moyen pour les tumeurs bénignes est de 47 ans et de 49 pour les tumeurs malignes [3]. Pour l'ensemble de nos patients, l’âge moyen était de 45 ans avec un pic de fréquence observé pour la 4^ème^ décade de vie. L’âge moyen des patients présentant des tumeurs bénignes était de 44 ans et ceux présentant des tumeurs malignes étaient de 50 ans. Dans notre série le sex-ratio (F/H) était 1,12. Il était de 1.34 pour les tumeurs bénignes et de 0.5 pour les tumeurs malignes. On note une nette prédominance masculine pour les tumeurs malignes. L'examen clinique peut mettre en évidence des signes fortement évocateurs de malignité: des douleurs (qui évoquent un envahissement cutané ou nerveux), une parésie faciale, une fixité, une croissance rapide [4]. La douleur est peu fréquente et considérée comme un facteur de mauvais pronostic [5]. Pour Lin, la douleur a été révélatrice chez 58% des tumeurs malignes [3]. Pour Poorten, la douleur est associée aux tumeurs malignes dans 25% des cas [6]. Dans notre série la douleur a été observée chez 27% des patients porteurs de tumeurs malignes et dans moins de 4% des tumeurs bénignes. Une parésie ou une paralysie faciale sont rapportées dans 12% à 20% des tumeurs malignes selon Spiro [7]. L'ancienneté d'une tuméfaction et la lenteur de sa croissance ne sont pas garantes de sa bénignité. Spiro rapporte 9% de tumeurs malignes évoluant depuis plus de 10 ans lors du diagnostic [7]. Dans notre série; Le délai moyen de consultation était de 24 mois pour les tumeurs bénignes et de 16 mois pour les tumeurs malignes. La paralysie faciale a été retrouvée dans 4 cas (20% des tumeurs malignes contre 2% des tumeurs bénignes). Des adénopathies cervicales sont notées dans 26% des cas des tumeurs malignes. La difficulté de diagnostic clinique justifie la réalisation d'examens complémentaires visant une approche diagnostique pour une meilleure prise en charge thérapeutique. Parmi les examens complémentaires, l'imagerie garde une place importante pour le diagnostic des tumeurs parotidiennes, l’échographie est un examen de réalisation simple, rapide et non invasif, elle permet le diagnostic des tumeurs parotidiennes dans près de 100% des cas [8, 9]. Néanmoins, cet examen est limité dans l'exploration du lobe profond et des tumeurs de grande taille [9, 10]. Certains signes échographiques tels le caractère hétérogène, mal limité et à contours flous de la tumeur peuvent être suspects de malignité [10, 11]. Ces signes ont été retrouvés dans 6 cas de notre série. Le scanner permet une évaluation précise du volume tumoral, l'exploration du lobe profond et une bonne analyse des structures osseuses, la détection d'adénopathies nécrotiques satellites plaide en faveur de la malignité [12]. L'IRM procure une excellente résolution anatomique et une information de nature tumorale fiable, elle permet de distinguer les lésions tissulaires des lésions kystiques et d’éliminer les lésions de contiguïté. Classiquement l'adénome pléomorphe se présente sous la forme d'une tumeur bien limitée, encapsulée avec un aspect polylobé caractéristique en hypo signal T1 et hyper signal T2 [2]. La cytoponction à l'aiguille fine, longtemps controversée, trouve de plus en plus sa place dans le diagnostic préopératoire des tumeurs parotidiennes, sa valeur diagnostique de nature tumorale est similaire voir supérieure à l'IRM et son coût demeure inférieur [13].

Le guidage échographique ou scannographique de la cytoponction est réservé aux tumeurs profondes ou non palpables [14]. On a recensé dans notre série 61 cas de tumeurs parotidiennes bénignes (80%) et 15 cas de tumeurs malignes (20%). l'adénome pléomorphe représente 65 à 75% des tumeurs parotidiennes et environ 80% des tumeurs épithéliales bénignes, dans notre série cette tumeur a représenté 61% des cas. La tumeur de Whartin est la deuxième tumeur parotidienne bénigne par ordre de fréquence et représente 5 à 15%, c'est une tumeur qui se développe le plus souvent au niveau du lobe superficiel [2]. Les tumeurs malignes représentent environ 20% des tumeurs parotidiennes [13], les carcinomes mucoépidermoïdes constituent avec les adénocarcinomes et les carcinomes adénoïdes kystiques les types histologiques les plus fréquents [13, 14], ces derniers sont caractérisés par une évolution lente et une tendance à l'envahissement nerveux [2, 13], les carcinomes à cellules acineuses sont relativement rares, ne représentant que 2 à 5% des tumeurs parotidiennes malignes et sont considérées comme des cancers de bas grade de malignité [2], les lymphomes malins des glandes salivaires représentent 5% des lymphomes extra ganglionnaires et sont dans la majorité des cas des lymphomes non hodgkiniens [2]. Pour notre série, les carcinomes muco-épidermoïdes étaient les plus fréquents dans 5 cas (33% des tumeurs malignes et 6% de toutes les tumeurs), un carcinome adénoïde kystique dans 4 cas, un carcinome à cellule claire dans 3 cas, un carcinome à cellule acineuse dans 2 cas, un cas de lymphome malin non hodgkinien et un cas d'adénocarcinome. Le traitement des tumeurs parotidiennes est basé essentiellement sur la chirurgie [2], les indications dépendent du siège, du volume tumoral et de la nature histologique guidée par un examen extemporané qui représente une étape incontournable du traitement [7]. En cas d'adénome pléomorphe, les auteurs préconisent une parotidectomie emportant la tumeur sans effraction capsulaire pour éviter les récidives, les petites tumeurs au dépend du lobe superficiel bénéficieront d'une parotidectomie exo faciale alors que pour les volumineuses tumeurs à développement endofacial une parotidectomie totale s'impose [2], certains auteurs optent pour une parotidectomie totale. Dans notre série, 37 patients (48%) ont subi une parotidectomie exo faciale. En cas de tumeur maligne, une parotidectomie totale est systématique. L'attitude vis-à-vis du nerf facial n'est pas codifiée, certains auteurs le conservent systématiquement même s'il est macroscopiquement envahi et complètent par une radiothérapie [2, 14]. Dans notre série la parotidectomie totale a été réalisée dans 52% des cas; le sacrifice du nerf facial a été effectué dans un seul cas. Concernant le curage ganglionnaire le problème se pose essentiellement pour les patients N0 cliniques et radiologiques, mais il est admis que le curage ganglionnaire est réservé pour les tumeurs T3 et T4 de haut grade de malignité, dans les autres cas un curage sélectif des zones II et III est réalisé, il sera complété en cas de positivité à l'examen extemporané par un curage fonctionnel classique [2, 14]. Pour les patients dont le N est différent de 0, un curage ganglionnaire fonctionnel est systématique [13, 14]. L'association radiothérapie-chirurgie améliore considérablement le pronostic des tumeurs malignes [14], les indications de la radiothérapie postopératoire communément admises actuellement sont: des limites d'exérèse envahies, les tumeurs de haut grade de malignité, l'extension tumorale extra glandulaire, l'atteinte du nerf facial, la présence d'adénopathie envahie et l'extension au lobe profond [8, 14]. La place de la chimiothérapie est réservée aux lymphomes et aux cancers très évolués [2], 12 de nos patients ont été adressés pour radiothérapie et 1 a subi une chimiothérapie pour un lymphome malin non hodgkinien. Les complications postopératoires sont non spécifiques à type d'infections et d'hématomes, ou bien spécifiques à type de paralysie faciale transitoire ou définitive, de fistules salivaires ou de syndrome de Frey [15]. Le pronostic des tumeurs parotidiennes dépend essentiellement du type histologique, il est généralement bon pour les tumeurs bénignes. Pour l'adénome pléomorphe le risque de récidive est estimé à moins de 2% [15]. Concernant les tumeurs malignes, les adénocarcinomes, les carcinomes épidermoïdes indifférenciés et les carcinomes ex-adénomes pléomorphes ont le plus mauvais pronostic avec possibilité de métastases à distance, alors que les carcinomes à cellules acineuses et les mucoépidermoïdes de bas grade ont un pronostic bien meilleur [14].

## Conclusion

Ce travail nous a permis d'exposer à travers l’étude des particularités épidemio-cliniques et paracliniques des 76 cas de tumeurs parotidiennes recensés dans notre étude; l'analyse de certains facteurs prédictifs de malignité de ces tumeurs. La malignité est évoquée cliniquement devant la douleur, la paralysie faciale, la fixité par rapport au plan superficiel ou profond et la présence d'adénopathie. L’échographie est l'examen de première intention à demander. Elle confirme la nature parotidienne de la tumeur et oriente vers sa bénignité ou sa malignité. L'IRM constitue, désormais, l'examen de choix dans l'exploration des masses tumorales parotidiennes avec une bonne valeur diagnostique de malignité ou de bénignité. La cytoponction à l'aiguille fine n'a pas de valeur que si elle est positive. La parotidectomie exploratrice avec examen anatomopathologique extemporané demeure la clé du diagnostic positif. Le traitement chirurgical est l'option de choix. La paralysie faciale est la complication la plus incommodante de cette chirurgie. Le pronostic des tumeurs malignes est tributaire de plusieurs facteurs, il semble considérablement amélioré en cas d'association radiothérapie-chirurgie.

### Etat des connaissance sur le sujet

- La pathologie tumorale de la glande parotide pose un problème diagnostique et thérapeutique.
- Une bonne analyse des facteurs prédictifs de malignité semble actuellement nécessaire en vue d'une meilleure planification thérapeutique.
- La cytoponction à l'aiguille fine n'a pas de valeur que si elle est positive.

### Contribution de notre étude à la connaissance

- Le sex-ratio (F/H) était 1,12 dans notre série, avec 1,34 pour les tumeurs bénignes et 0,5 pour les tumeurs malignes.
- Dans notre série 80% de tumeurs parotidiennes étaient bénignes et 20% malignes.
- Les prédicteurs cliniques de malignité dans notre série sont la douleur, la paralysie faciale, la fixité et la présence d'adénopathie.
